# Determination and prioritization of factors affecting the occurrence of needle stick injuries among healthcare workers using techniques of Delphi and fuzzy analytical hierarchy process (FAHP)

**DOI:** 10.1186/s12889-023-16969-x

**Published:** 2023-10-16

**Authors:** Seyed Mahdi Mousavi, Saeid Yazdanirad, Sara Althubiti, Masoud Askari Majdabadi, Faranak Najarian, Parvin Sepehr

**Affiliations:** 1 Department of Occupational Health and Safety Engineering, School of Health, Isfahan University of Medical Sciences, Isfahan, Iran; 2 Social Determinants of Health Research Center, Shahrekord University of Medical Sciences, Shahrekord, Iran; 3 School of Health, Shahrekord University of Medical Sciences, Shahrekord, Iran; 4 Department of Computer Science, College of Computer and Information Sciences, Majmaah University, Al-Majmaah, Saudi Arabia; 5 Department of Occupational Health, School of Public Health, Tehran University of Medical Sciences, Tehran, Iran; 6 Department of Occupational Health, School of Public Health, Shahid Beheshti University of Medical Sciences, Tehran, Iran

**Keywords:** Needle stick injuries, Needlestick, Risk factors, Healthcare workers, Delphi, Fuzzy hierarchical analysis

## Abstract

**Introduction:**

Needlestick injuries (NSIs) are a major hazard in the workplace for healthcare workers. To prevent these injuries, it is essential to determine the important factors affecting the occurrence of them. This study aimed to identify, classify and prioritize these factors using techniques of Delphi and fuzzy analytical hierarchy process (FAHP).

**Methods:**

This descriptive-analytical study was conducted in 2022. Firstly, the factors affecting the occurrence of needlestick injuries were identified by the literature review. Moreover, the Delphi technique was used to identify the factors. 20 experts (physicians, nurses, and occupational health experts) participated in the steps of the Delphi method. Then, these factors were grouped into six groups. In the next step, the fuzzy analytical hierarchy process (FAHP) was applied to prioritize the factors. For this purpose, the pairwise comparison questionnaire was designed and filled out by 20 experts. Finally, data were analyzed using MATLAB software (version 2018a).

**Results:**

42 factors (31 factors extracted from the literature review and 11 factors obtained from the Delphi technique) were identified in this study. These factors were categorized into six groups. Based on the results, the relative weight of non-demographic personal factors, tool and technology factors, job factors, organizational factors, demographic personal factors, and environmental factors were computed by 0.200, 0.185, 0.184, 0.157, 0.142, and 0.133, respectively.

**Conclusion:**

These results determined the importance of the factors affecting the occurrence of needlestick injuries. These findings can be useful for planning preventive measures.

## Introduction

There are various hazards in occupational environments [1]. Needlestick injuries (NSIs) are a major hazard in the workplace for healthcare workers [2]. The NSIs are defined as skin tissue damage caused by the needle, broken syringe, and other sharp instruments [3]. If the syringe is contaminated with blood or other secretions of the patient, dangerous diseases such as hepatitis B (HBV) and hepatitis C (HCV), and human immunodeficiency virus (HIV) can be transmitted to the person [4]. According to estimates, between 32.4 and 44.5% of healthcare workers experience at least one needle stick or sharps injury per year [5]. Various studies have identified risk factors affecting the occurrence of NSIs [6]. In a study performed by Bazie, these risk factors have been divided into four main groups, including socio-demographic factors, organizational factors, environmental factors, and behavioral factors [7]. Socio-demographic factors include gender, age, education level, and work experience [8]. Organizational factors are shift work, safety climate, job stress, and safety culture [9]. Environmental factors include noise, lighting, and heat stress, and behavioral factors are skill in injection and belief in the dangerous nature of NSIs [10, 11]. Also, Ghasemi et al. concluded that color vision defects, abnormal heterophoria, and decreasing contrast sensitivity are significantly associated with the occurrence of needlestick injuries [12]. Ghimire et al. also observed that there are significant relationships between factors of age, depression, social problems, alcohol consumption, and sleep quality and the occurrence of needlestick injuries among healthcare workers [13]. The results of a study conducted by Jahangiri et al. showed that gender, working hours per week, and work shifts per month can influence the occurrence of needlestick injuries [14].

These factors have been dispersedly introduced in various studies. Therefore, conducting a literature review and utilizing the Delphi technique can be helpful for the comprehensive identification of them. The Delphi technique is a systematic and qualitative method for collecting experts’ opinions [15]. This technique can provide a strong and robust consensus of opinions [16]. Other advantages of this technique include high flexibility for various approaches, useability in different disciplines, and the ability to open discussion. This technique has been widely used in various fields, including medical, engineering, and health sciences, for identifying the factors affecting a specific phenomenon [17].

Given that the importance of all risk factors is not equal, multi-criteria decision-making techniques may be necessary to prioritize them [18]. The Analytical Hierarchy Process (AHP) is one of the most well-known multi-criteria decision-making techniques. The AHP helps decision-makers to determine priorities based on their goals, knowledge, and experience. However, decision-makers may have difficulty expressing their judgments due to the fuzzy nature and uncertainty of the factors. To solve this problem, the Fuzzy Analytical Hierarchy Process (FAHP) method has been developed [18]. The FAHP method has been applied in various fields, including medical and health sciences. For example, Rajabi et al. used this technique to prioritize occupational stressors among nurses [19]. Hosseini et al. applied this method to rank the factors affecting field choice among nursing students [20]. Similarly, Kimiafar et al. used the FAHP method to prioritize factors influencing nurses’ satisfaction from hospital information systems [21].

As previously mentioned, various factors can affect the occurrence of needlestick injuries. Identifying, classifying, and prioritizing these factors can be helpful in preventing these injuries. However, previous studies have dispersedly introduced some of them. Therefore, it is required that a comprehensive study is performed on this issue. The present study aimed to comprehensively identify, classify and prioritize the factors affecting the occurrence of needlestick injuries using techniques of Delphi and fuzzy analytical hierarchy process (FAHP).

## Methods

This descriptive-analytical study was conducted in three stages as follows.

### Identifying the factors affecting the occurrence of needlestick injuries

At this stage, firstly, the non-systematic literature review was performed in reliable databases, such as ISI, PubMed, and Scopus. The keywords were selected based on the initial search and researchers’ opinions, and those were divided into two groups. The strategy search was a combination of keywords from the first and second groups. The keywords of the first group consisted of impact, effect, factor, risk factor, agent, item, relationship, prediction, association, and associated. The keywords of the second group included needlestick, needle stick, sharp injury, and needle-stick. The cohort, case-control, retrospective, and cross-sectional studies in the English language without time restrictions were entered into the review. In the next step, irrelevant studies and articles without inclusion criteria were removed. Then, two independent reviewers (S.Y and S.P) carefully reviewed the articles and extracted the factors. The search strategy done in PubMed, as an example, is shown below.

(((((((((((Impact[Title/Abstract]) OR (effect[Title/Abstract])) OR (factor[Title/Abstract])) OR (“risk factor“[Title/Abstract])) OR (agent[Title/Abstract])) OR (item[Title/Abstract])) OR (relationship[Title/Abstract])) OR (prediction[Title/Abstract])) OR association[Title/Abstract])) OR (associated[Title/Abstract]) AND (english[Filter])) AND (((((Needlestick[Title/Abstract]) OR (needle stick[Title/Abstract])) OR (sharp injury[Title/Abstract])) OR (needle-stick[Title/Abstract]) AND (english[Filter]))

Delphi technique in two rounds is used to identify other factors. 20 experts (physicians, nurses, and occupational health experts) performed the steps of the Delphi method. The inclusion criteria included having a career length greater than two years and having a history of educational and research activities on needlestick or having work experience in hospital wards. The exclusion criterion also included unwillingness to cooperate in the study and having illogical and inconsistent opinions. For conducting the Delphi technique, the list of classified factors was sent to experts and they were asked to introduce other factors in addition to factors identified by the literature review. Also, they were asked to state their opinions on the classification of factors. In the next step, the answers were gathered and analyzed. Then, the proposed factors were added to the list. After that, this list is again sent to experts and they were asked to express their opinion on the factors. Finally, the answers were collected and analyzed, and the list was revised.

### Classifying the identified factors

In this step, all identified factors were classified into six groups based on the balance theory of job design. Based on this theory, a working system consists of five elements, including individual, task, tools and technology, organization, and environment. The imbalance between these elements can produce a stress load [22]. In the present study, this theory was used to categorize the factors affecting the occurrence of needlestick injuries. In the present study, those included demographic and non-demographic personal factors, job factors, tools and technology factors, organizational factors, and environmental factors, respectively. Also, in this stage, irrelevant and duplicate factors are removed.

### Prioritizing the identified factors

At this stage, the relative weight of the factors was computed using the fuzzy analytical hierarchy process (FAHP) method. For this purpose, a hierarchical structure was first drawn. Then, tables of pairwise comparison were prepared and sent to 20 experts (professors and hospital experts). They compared the factors in terms of relative importance in the occurrence of needlestick injuries. After that, the linguistic words were converted into triangular fuzzy numbers, as presented in Table 1. To calculate the weight of the factors, the fuzzy method developed by Chang et al. was used [18]. The computational steps of this method have been presented as follows.

**Table 1:** Linguistic words and their synonymous triangular fuzzy numbers

Linguistic word	Fuzzy number scale	Fuzzy numbers
Equally important	1	(1,1,3)
Slightly more important	2	(1,3,5)
More important	3	(3,5,7)
Much more important	4	(5,7,9)
Extremely more important	5	(7,9,11)

#### Step 1: forming paired comparison matrix

The paired comparison was performed by the decision matrix (Eq. 1).

1 $${\rm{\tilde A = }}\left[ {\begin{array}{*{20}{c}}{\rm{1}}&{{{\rm{M}}_{{\rm{12}}}}}&{\rm{ \ldots }}&{{{\rm{M}}_{{\rm{1n}}}}}\\{{{\rm{M}}_{{\rm{21}}}}}&{\rm{1}}&{\rm{ \ldots }}&{{{\rm{M}}_{{\rm{2n}}}}}\\{\rm{M}}&{\rm{M}}&{\rm{O}}&{\rm{M}}\\{{{\rm{M}}_{{\rm{n1}}}}}&{{{\rm{M}}_{{\rm{n2}}}}}&{\rm{ \ldots }}&{\rm{1}}\end{array}} \right]$$

#### Step 2: calculating Si

Si is the triangular fuzzy number related to the relative weight of each criterion. It is computed by Eq. 2.

2 $${S}_{i}= \sum _{j=1}^{m}{M}_{gi}^{J} \times {\left[\sum _{i=1}^{n}\sum _{j=1}^{m}{M}_{gi}^{J}\right]}^{-1}$$

Where i, j and ${M}_{gi}^{j}$are the column number, row number, and fuzzy numbers of the paired matrix, respectively. $\sum _{j=1}^{m}{M}_{gi}^{J}$, $\sum _{i=1}^{n}\sum _{j=1}^{m}{M}_{gi}^{i}$ and ${\left[\sum _{i=1}^{n}\sum _{j=1}^{m}{M}_{gi}^{i}\right]}^{-1}$ were estimated by the following equations.

3 $$\sum _{j=1}^{m}{M}_{gi}^{j}= \left(\sum _{j=1}^{m}{l}_{j}\sum _{j=1}^{m}{m}_{j}\sum _{j=1}^{m}{u}_{j}\right)$$

4 $$\sum _{i=1}^{n}\sum _{j=1}^{m}{M}_{gi}^{j}= \left(\sum _{i=1}^{m}{l}_{i}\sum _{i=1}^{m}{m}_{i}\sum _{i=1}^{m}{u}_{i}\right)$$

5 $${\left[\sum _{i=1}^{n}\sum _{j=1}^{m}{M}_{gi}^{j}\right]}^{-1}= \left(\frac{1}{\sum _{i=1}^{m}{u}_{i}} \frac{1}{\sum _{i=1}^{m}{m}_{i}} \frac{1}{\sum _{i=1}^{m}{l}_{i}}\right)$$

#### Step 3: calculating the possibility degree

If S_1_ = (l_1_, m_1_, u_1_) and S_2_ = (l_2_, m_2_, u_2_) are two triangular fuzzy numbers, the degree of possibility of S_2_ ≥ S_1_ is described by the following equations.

6 $$V \left({S}_{2}\ge {S}_{1}\right)=hgt \left({S}_{2}\cap {S}_{1}\right)= {}_{{S}_{1}}\left(d\right)= \left\{\begin{array}{c}1 if {m}_{2}\ge {m}_{1} \\ 0 if {l}_{1}\ge {u}_{2}\\ \frac{{l}_{1}-{u}_{1}}{\left({m}_{2}-{u}_{2}\right)- \left({m}_{1}-{l}_{1}\right) } otherwise\end{array}\right.$$

On the other hand, the possibility degree of a triangular fuzzy number relative to k triangular fuzzy numbers was calculated by the following equations.

7 $$\begin{array}{l}V\left( {{S_2} \ge {S_1}{S_2} \ldots {S_K}} \right) = V\left[ {\left( {S \ge {S_1}} \right)\,and\,\left( {S \ge {S_2}} \right)\,and\, \ldots \,and\,\left( {S \ge {S_k}} \right)} \right]\\\,\,\,\,\,\,\,\,\,\,\, = MinV\left( {S \ge {S_i}} \right).i = 1.2.3. \ldots .k\end{array}$$

#### Step 4: calculating criteria weight

Equation 8 was applied to calculate the weight vector of criteria in the paired matrix.

8 $${d}^{{\prime }}\left({A}_{i}\right)=Min V \left({S}_{i}\ge {S}_{k}\right) k=1. 2. \dots . n . k\ne i$$

Thus, the non-normal weight vector will be as follows:

9 $${W}^{{\prime }}= {\left({d}^{{\prime }}\left({A}_{1}\right). {d}^{{\prime }}\left({A}_{2}\right). \dots .{d}^{{\prime }}\left({A}_{n}\right)\right)}^{T} {A}_{i }\left(i=1. 2. \dots . n\right)$$

#### Step 5: calculating normal weight

Equation 10 is used to compute the normal weight by normalizing the non-normal weight vector obtained from the previous step.

10 $$W= {\left(d\left({A}_{1}\right). d\left({A}_{2}\right). \dots .d\left({A}_{n}\right)\right)}^{T}$$

Moreover, the geometric mean value was applied to combine the opinions of experts (Eq. 11).

11 $${a}_{ij}= {\left(\prod _{K=1}^{K}{A}_{ijk}\right)}^{\raisebox{1ex}{$1$}\!\left/ \!\raisebox{-1ex}{$K$}\right.} K=1. 2. \dots . K$$

#### Step 6: calculating consistency index

At this step, the consistency index was computed by the Gogus and Boucher method to ensure the reliability of the findings. Based on the results, the consistency index of all matrices was lower than 0.1. Therefore, the obtained results were reliable [18].

### Data analysis

SPSS software version 22 was used for the descriptive analysis of data. To prioritize the factors, the calculations were performed in MATLAB software (version 2018a) based on the Chang method [18]. Also, the consistency index was computed by the Gogus and Boucher method in this software.

## Result

**Fig. 1:**
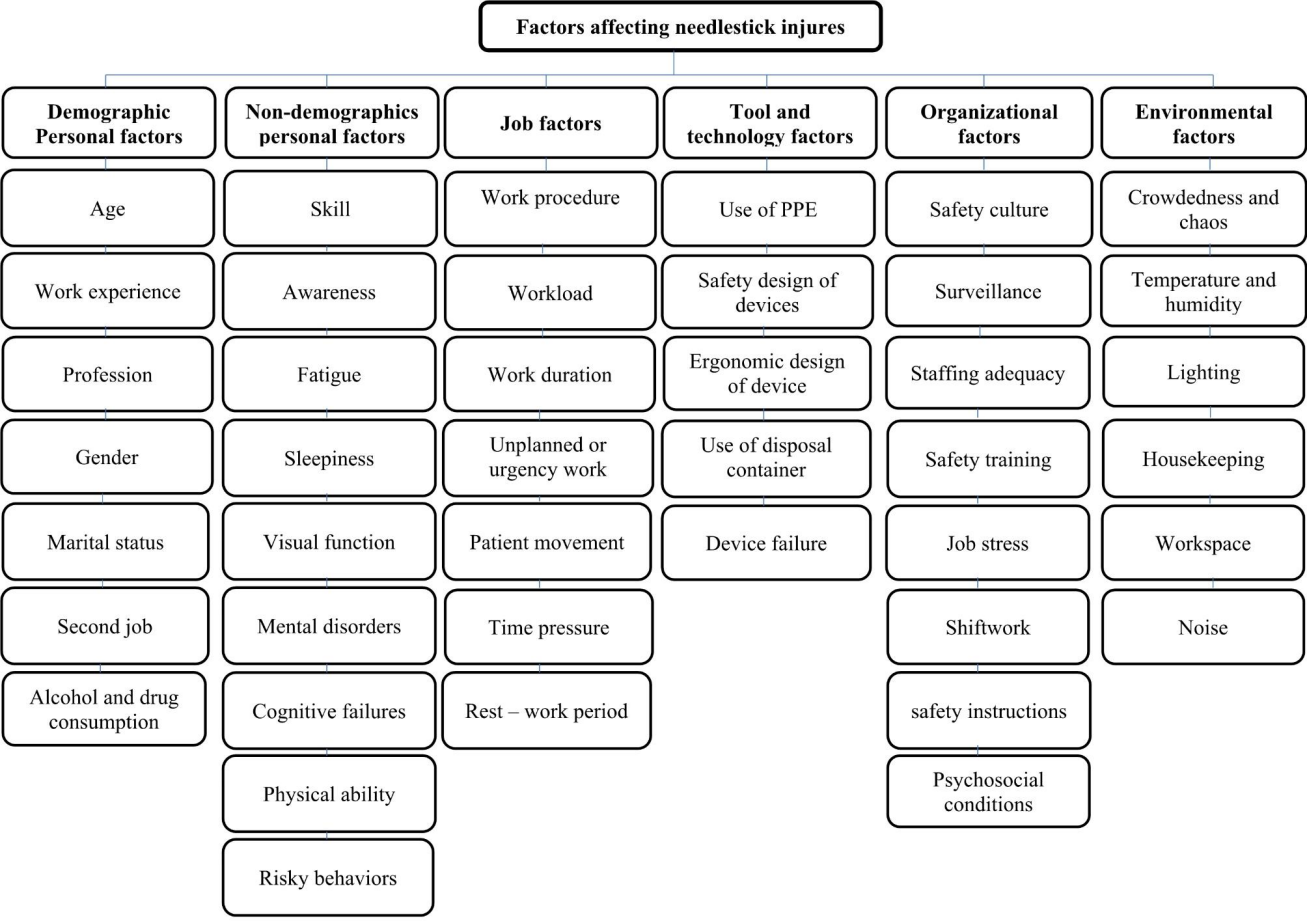
The hierarchical structure of the identified factors

In the literature review step, 303 articles were found. Of these articles, 92 repetitive studies were removed and 211 articles were studied. Then, 80 factors were extracted from these studies. Of them, 39 irrelevant and duplicate factors are removed and 31 factors remained. In addition to these factors, 11 factors were identified by the Delphi method. The Delphi survey was sent to 30 experts in the first round. Of them, 24 persons (80.00%) answered. Then, the corrected survey was again sent to 24 experts in the second round. Of them, 20 persons (83.33%) answered. In total, 42 factors were identified and classified into six groups. Table 2 represents the identified factors affecting the occurrence of needlestick injuries. Moreover, Fig. 1 shows the hierarchical structure of these factors.

**Table 2:** The identified factors affecting the occurrence of the needlestick injuries

Demographic personal factors	Non- demographic personal factors	Job factors	Tool and technology factors	Organizational factors	Environmental factors
Age [23, 24]	Skill [25, 26]	Work procedure [25, 27]	Use of Personal protective equipment [28, 29]	Safety culture [30, 31]	Crowdedness and chaos [32]
Work experience [7, 33]	Awareness [34, 35]	Workload [36, 37]	Safety design of devices [38, 39]	Surveillance [40]	Temperature and humidity [41]
Profession [42, 43]	Fatigue [44, 45]	Work duration [46, 47]	Ergonomic design of device [48]	Staffing adequacy [34, 49]	Lighting [48, 50]
Gender [51, 52]	Sleepiness [53, 54]	Unplanned or urgency work [55]	Use of disposal container [7, 56]	Safety training [27, 57]	Housekeeping^*^
Marital status [46, 58]	Visual function [12]	Patient movement [36]	Device failure [36]	Job stress [58, 59]	Workspace^*^
Second job^*^	Mental disorders^*^	Time pressure^*^		Shiftwork [60, 61]	Noise^*^
Alcohol and drug consumption^*^	Cognitive failures^*^	Rest – work period^*^		safety instructions [62]	
	Physical ability^*^			Psychosocial conditions [63, 64]	
	Risky behaviors^*^				

* These factors were identified by the Delphi method

Table 3 represents the relative weight of the groups. Based on the results, non-demographic personal factors had the highest relative weight among groups (0.200). The other groups included tool and technology factors (0.185), job factors (0.184), organizational factors (0.157), demographic personal factors (0.142), and environmental factors (0.133), respectively.

**Table 3:** The relative weight of the groups

Group	Fuzzy weight			Final weight
	L	M	U	
Demographic personal factors	0.123	0.252	0.314	0.142
Non- demographic personal factors	0.111	0.143	0.214	0.200
Job factors	0.131	0.178	0.312	0.184
Tool and technology factors	0.127	0.199	0.301	0.185
Organizational factors	0.132	0.228	0.316	0.157
Environmental factors	0.139	0.231	0.211	0.133

Table 4 reports the relative weight of the factors. Among demographic personal factors, the profession had the highest relative weight (0.208). The other factors included alcohol and drug consumption (0.184), work experience (0.172), age (0.125), gender (0.112), second job (0.111), and marital status (0.088), respectively. Of the non-demographic personal factors, skill level obtained the highest relative weight (0.114). The other factors included cognitive failures (0.113), fatigue (0.112), risky behaviors (0.111), sleepiness (0.108), visual function (0.093), awareness (0.092), mental disorders (0.089), and physical ability (0.084), respectively. Among job factors, the highest relative weight was related to the workload (0.171). The other factors included work procedure (0.167), time pressure (0.141), unplanned or urgency work (0.135), patient movement (0.133), time pressure (0.132), rest–work period (0.121), and work duration (0.121), respectively. Of tool and technology factors, the use of personal protective equipment had the highest relative weight (0.222). The other factors consisted of the safety design of devices (0.205), use of disposal containers (0.199), device failure (0.197), and ergonomic design of devices (0.177), respectively. Among organizational factors, the highest relative weight belonged to job stress (0.143). The other factors included shiftwork (0.132), psychosocial conditions (0.128), staffing adequacy (0.127), safety training (0.123), safety instructions (0.117), safety culture (0.116), and surveillance (0.115), respectively. Of environmental factors, crowdedness and chaos (0.213) obtained the highest relative weight. The other factors were lighting (0.207), housekeeping (0.204), workspace (0.188), noise (0.185), and temperature and humidity (0.177), respectively.

**Table 4:** The relative weight of the factors

Group	Factor	Fuzzy weight			Final weight
		L	M	U	
Demographic personal factors	Profession	0.125	0.176	0.238	0.208
	Alcohol and drug consumption	0.120	0.152	0.227	0.184
	Work experience	0.119	0.151	0.221	0.172
	Age	0.117	0.144	0.211	0.125
	Gender	0.115	0.123	0.209	0.112
	s job	0.113	0.113	0.190	0.111
	Marital status	0.101	0.111	0.188	0.088
Non-demographic personal factors	Skill level	0.142	0.189	0.261	0.114
	Cognitive failures	0.162	0.182	0.211	0.113
	Fatigue	0.118	0.211	0.260	0.112
	Risky behaviors	0.136	0.186	0.225	0.111
	Sleepiness	0.141	0.153	0.198	0.108
	Visual function	0.156	0.162	0.211	0.093
	Awareness	0.139	0.145	0.209	0.092
	Mental disorders	0.123	0.136	0.196	0.089
	Physical ability	0.115	0.166	0.199	0.084
Job factors	Workload	0.116	0.132	0.211	0.171
	Work procedure	0.132	0.151	0.176	0.167
	Time pressure	0.163	0.181	0.222	0.141
	Unplanned or urgency work	0.142	0.178	0.231	0.135
	Patient movement	0.131	0.184	0.201	0.133
	Rest – work period	0.156	0.175	0.200	0.132
	Work duration	0.147	0.168	0.217	0.121
Tool and technology factors	Use of personal protective equipment	0.181	0.196	0.221	0.222
	Safety design of devices	0.152	0.171	0.195	0.205
	Use of disposal containers	0.171	0.185	0.210	0.199
	Device failure	0.146	0.165	0.211	0.197
	Ergonomic design of device	0.139	0.173	0.197	0.177
Organizational factors	Job stress	0.131	0.191	0.221	0.143
	Shiftwork	0.141	0.185	0.201	0.132
	Psychosocial conditions	0.121	0.175	0.211	0.128
	Staffing adequacy	0.142	0.168	0.198	0.127
	Safety training	0.157	0.189	0.203	0.123
	safety instructions	0.134	0.153	0.178	0.117
	Safety culture	0.137	0.149	0.196	0.116
	Surveillance	0.131	0.154	0.175	0.115
Environmental factors	Crowdedness and chaos	0.142	0.189	0.220	0.213
	Lighting	0.139	0.182	0.210	0.207
	Housekeeping	0.137	0.179	0.208	0.204
	Workspace	0.135	0.165	0.193	0.188
	Noise	0.130	0.150	0.190	0.185
	Temperature and humidity	0.127	0.141	0.188	0.177

## Discussion

Based on the results, non-demographic personal factors had the first priority among the groups. Of non-demographic personal factors, the highest priorities were related to skill level, cognitive failures, and fatigue.

Skill is an important factor in determining the risk of needlestick injuries. People with high skill in injection have fewer proneness in occurring needlestick injuries. Also, skill level can play an important role in reducing human error. This finding is consistent with the results of the studies conducted by Kwanzaa et al. and Al Qadire et al. The results of these studies showed that the increasing level of skill decreases the occurrence of needlestick injuries [65]. The results of a study performed by Clarke et al. also indicated that low skill levels significantly increase the occurrence of needlestick injuries among nurses (odds ratio = 1.23) [66, 67]. Moreover, Ali et al. concluded that low skill level is one of the most important reasons in occurring of needlestick injuries among students [68].

Cognitive failures, as another important factor, are defects in perception, memory, and motor functioning. Therefore, it could be stated that cognitive failures affect the ability of persons in performing their tasks. Mohammady et al. also observed that cognitive failure is one of the most important predictors of patient safety [69]. It may be because these failures cause disruptions in the decision-making and performance of healthcare workers [69]. Moreover, the results of a study performed by Yousef Zade et al. indicated that there is a significant relationship between cognitive failures and human errors among nurses, which can impress on patient and personnel safety [70].

In the present study, fatigue was introduced as another important factor among non-demographic personal factors. Fatigue is associated with many negative outcomes and consequences. It is an unpleasant mental feeling, that influences individual performance [71]. Fatigue can occur because of long working hours and insufficient refreshing time. In a study performed by Sharma et al., 50.4% of the participants introduced fatigue as one of the important reasons for their needlestick injuries [72]. The results of a study conducted by Akbari et al. showed that fatigue is the most important predictor of needlestick injuries [73]. Fatigue can lead to increasing cognitive failures among healthcare workers and thereby make needlestick injuries [73]. Therefore, considering the findings of the previous studies, the results of the present study can be logical.

In the present study, tool and technology factors obtained the second rank. Of these factors, the highest priorities belonged to the use of personal protective equipment and the safety design of devices, respectively.

Access to personal protective equipment and the correct use of this equipment play a great role in reducing the occurrence of needlestick injuries among healthcare personnel. The results of a study carried out by Semere Reda et al. revealed inadequate access to personal protective equipment can significantly increase the likelihood of occupational exposure to blood among healthcare workers (odds ratio = 3.88) [74]. Dulon et al. concluded that access to personal protective equipment and correct use of this equipment can substantially reduce the occurrence of needlestick injuries [36]. However, there are challenges with the use of this equipment [75]. Therefore, educational interventions can be necessary to change the attitude of people [75].

In this study, the third rank was related to the job factors. Of these factors, workload, work procedure, and time pressure had the highest priorities, respectively.

The workload is defined as the amount of work to be done by a person in a time period. Workload has five aspects, including mental demand, physical demand, temporal demand, performance effort, and frustration [76]. The increasing workload can be associated with increasing fatigue and human error among healthcare workers. Hosseinabadi et al. also observed that a heavy workload can increase the occurrence of needlestick injuries by 35% [76]. Yusef Zadeh et al. concluded that there is a significant correlation between mental workload and cognitive failures among healthcare workers [70].

In addition, in the current study, work procedure and time pressure were introduced as other important factors. It is clear that weak procedures can be associated with increasing errors and exposures. Also, time limitations and pressure under critical conditions can decrease the mental ability of humans. The results of a study performed by Hoboubi et al. indicated that there is a significant relationship between temporal pressure and needlestick injuries [77].

Based on the results, organizational factors obtained the fourth rank. Of these factors, the highest priorities were related to job stress, shiftwork, and psychosocial conditions, respectively.

High job stress obtained the first rank among organizational factors. It may be because healthcare personnel have high stress due to the nature of their jobs, which disrupts their concentration and performance. The results of a study conducted by Asadi Fakhr et al. showed that 70% of nurses occupied in operating rooms reported high job stress [78]. Abadiga et al. also concluded that high job stress was significantly associated with needlestick and sharp injury (odds ratio = 1.93) [79]. The six main sources of job stress include job nature, role, communications, job development, organizational structure, and work-family interaction [80, 81]. Also, there is a substantial relationship between occupational stress and job workload. The results of a study performed by Sharif Nia et al. indicated that job stress due to high workload can increase the occurrence of occupational injuries, such as needlestick injuries [82]. Moreover, d’Ettorre et al. concluded that the implementation of interventions for stress management could significantly decrease the occurrence of NSIs (odds ratio of 0.60) [83].

In the present study, shift work was introduced as another organizational factor. Shift work is defined as prolonged shifts or rotating shift schedules. Trinkoff et al. concluded that there is a significant association between the schedule of shift work and occurrence of the needlestick injuries among nurses (relative risk = 1.63) [84]. Moreover, Trinkoff, in another study, observed that the highest risk of injuries was related to nurses with work time higher than 13 h and rotating shift work [85]. Also, the results of a study conducted by Canini et al. showed that night shift work or a combination of day and night shift work could significantly increase the occurrence of needlestick injuries among nurses (odds ratio = 2.77) [86]. During the night shift work, the occurrence of needlestick injuries may be increased because of fatigue, sleepiness, and insufficient concentration. Moreover, shift work can be associated with adverse health effects, such as overweight, cardiovascular diseases, and social life disruption. This situation can lead to a decrease in work efficiency and an increase in human error [87, 88].

Based on the results, demographic personal factors had the fifth rank. Of these factors, the highest priorities belonged to profession, alcohol and drug consumption, and work experience, respectively.

The prevalence of needlestick injuries is higher among some professions because of the nature of their work. In hospitals and medical centers, nurses have the highest statistics of needlestick injuries. It may be because most of the dangerous activities, such as drug injection and blood collection, are performed by nurses. The results of a systematic review conducted by Motaarefi et al. revealed that the highest occurrence of needlestick injuries occurred among nurses [6]. Alfulayw et al. also observed that the highest prevalence of needlestick injuries was related to nurses (52.2%) and physicians (24.9%) [2].

Alcohol and drug consumption, as another major factor, is one of the most important causes of occupational accidents in various workplaces, such as medical centers, industries, and road traffic. It is clear that alcohol and drugs can affect the normal function of the central nervous system in humans and create consequences such as reducing reaction time, reducing accuracy and concentration, and increasing accident susceptibility [89]. Searby et al. introduced occupational stress as one of the main reasons for alcohol and drug consumption among nurses [15].

In the present study, environmental factors had the sixth rank. Of these factors, the highest priorities were assigned to crowdedness and chaos, lighting, and housekeeping, respectively.

Crowdedness and chaos can be associated with distraction and stress, and it can lead to increasing human error. In the study of Kazemi Galougahi, crowdedness and chaos were introduced as the most important factor affecting the occurrence of needlestick injuries among healthcare workers [90].

Lighting is another important factor in the current study. The results of the epidemiological studies show that poor lighting is one of the important factors influencing the occurrence of needlestick injuries. Lighting can affect the employees’ vision, fatigue, mental pressure, and work efficiency [91]. These results are consistent with the findings of the present study.

As a limitation, the internal relationships among factors have been not considered in this study. Therefore, it is suggested that other multi-criteria decision-making methods, such as the DEMATEL and ANP methods, are used to investigate the internal relationship between factors in the next studies.

## Conclusion

In total, the results showed that non-demographic personal factors had the highest importance in occurring needlestick injuries. Other groups included tools and technology factors, job factors, organizational factors, demographic personal factors, and environmental factors, respectively. These findings can be useful for planning preventive strategies to reduce the occurrence of needlestick injuries in hospitals. It is recommended that the factors with high importance are first taken attention. Controlling these factors can substantially decrease the occurrence of needlestick injuries among healthcare workers. Therefore, increasing skill level, reducing cognitive failure, reducing fatigue, increasing access to personal protective equipment, reducing mental workload, reducing occupational stress, optimizing shift work, reducing crowdedness and chaos, and improving the lighting can significantly decrease the statistics of the needlestick injuries.

## Data Availability

The datasets used and/or analyzed during the current study are available from the corresponding author on reasonable request.
